# Population Differences and Host Species Predict Variation in the Diversity of Host-Associated Microbes in *Hydra*

**DOI:** 10.3389/fmicb.2022.799333

**Published:** 2022-03-03

**Authors:** Jan Taubenheim, Máté Miklós, Jácint Tökölyi, Sebastian Fraune

**Affiliations:** ^1^Research Group Medical Systems Biology, Institute for Experimental Medicine, Medical Systems Biology, University Hospital Kiel, Kiel, Germany; ^2^Institut für Zoologie und Organismische Interaktionen, Heinrich-Heine Universität Düsseldorf, Düsseldorf, Germany; ^3^MTA-DE “Momentum” Ecology, Evolution and Developmental Biology Research Group, Department of Evolutionary Zoology, University of Debrecen, Debrecen, Hungary; ^4^Juhász-Nagy Pál Doctoral School of Biology and Environmental Sciences, University of Debrecen, Debrecen, Hungary

**Keywords:** determinants of microbial community, holobiont assembly, metaorganism, ecology of microbial communities, host-microbe balance/interaction

## Abstract

Most animals co-exist with diverse host-associated microbial organisms that often form complex communities varying between individuals, habitats, species and higher taxonomic levels. Factors driving variation in the diversity of host-associated microbes are complex and still poorly understood. Here, we describe the bacterial composition of field-collected *Hydra*, a freshwater cnidarian that forms stable associations with microbial species in the laboratory and displays complex interactions with components of the microbiota. We sampled *Hydra* polyps from 21 Central European water bodies and identified bacterial taxa through 16S rRNA sequencing. We asked whether diversity and taxonomic composition of host-associated bacteria depends on sampling location, habitat type, host species or host reproductive mode (sexual vs. asexual). Bacterial diversity was most strongly explained by sampling location, suggesting that the source environment plays an important role in the assembly of bacterial communities associated with *Hydra* polyps. We also found significant differences between host species in their bacterial composition that partly mirrored variations observed in lab strains. Furthermore, we detected a minor effect of host reproductive mode on bacterial diversity. Overall, our results suggest that extrinsic (habitat identity) factors predict the diversity of host-associated bacterial communities more strongly than intrinsic (species identity) factors, however, only a combination of both factors determines microbiota composition in *Hydra*.

## Introduction

The microbial organisms that animals coexist with [collectively known as the host associated microbiota (Berg et al., 2020)] have an increasingly recognized effect on their host (McFall-Ngai et al., 2013). The microbiota often influences host health (Shreiner et al., 2015; Rathje et al., 2020), behavior (Murillo-Rincon et al., 2017; Vuong et al., 2017), and in some cases it was shown to affect fitness-determining traits of the host, such as its growth rate, reproduction, survival and aging (Shin et al., 2011; Sison-Mangus et al., 2015; Gould et al., 2018; Popkes and Valenzano, 2020).

One of the most important aspects of the microbiota is its composition and diversity. Diversity affects the number and type of interactions that can be present in a microbial community (Coyte et al., 2015) and influence tripartite interactions between the host, microbiota and pathogens (Fraune et al., 2015; Harrison et al., 2019). Furthermore, diversity can affect the stability of microbial communities and their resilience to perturbation (Vieira-Silva et al., 2016) and reduction in diversity often leads to dysbiosis in response to environmental stressors or host diet changes (Infante-Villamil et al., 2020). Therefore, understanding the factors that determine microbial diversity in natural populations is key to explain variation in host physiology.

In natural populations, the diversity and composition of host-associated microbes is affected by several factors, such as host diet (Sullam et al., 2015; Lutz et al., 2019; Youngblut et al., 2019), host genotype and population genetic diversity (Sullam et al., 2015; Griffiths et al., 2018; Webster et al., 2018), features of the habitat, such as salinity (Schmidt et al., 2015; Mortzfeld et al., 2016), water temperature (Vargas et al., 2021), or pH (Sylvain et al., 2016), as well as geography (Llewellyn et al., 2016; Mortzfeld et al., 2016) and climate (Kueneman et al., 2019; Woodhams et al., 2020). However, the importance of these factors is only recently starting to be revealed and the mechanisms driving variation in microbial diversity are still poorly understood. Crucially, accumulating evidence indicates that the relative importance of these factors might vary among taxa. For instance, host evolutionary history was found to be a strong driver of microbial diversity in a range of animal groups from sponges and corals to various vertebrate groups (Franzenburg et al., 2013; Easson and Thacker, 2014; Pollock et al., 2018; Youngblut et al., 2019). Conversely, environmental factors appear to be predominant drivers in other taxa (such as freshwater zooplankton), without any evidence for phylosymbiosis (Eckert et al., 2021).

The freshwater cnidarian *Hydra* coexists with a rich diversity of host-associated microbes. These microbes colonize external epithelial surfaces (Fraune et al., 2015; Deines and Bosch, 2016), inhabit intercellular spaces (Rathje et al., 2020), and in some species can even occur inside cells as endosymbionts (Fraune and Bosch, 2007). The *Hydra* microbiota has diverse effects on the host. Animals with their microbiota experimentally removed have reduced movement and contractility (Murillo-Rincon et al., 2017) and impaired ability to reproduce asexually (Rahat and Dimentman, 1982). Interactions between microbial components influence pattern formation (Taubenheim et al., 2020), and can induce tumor development (Rathje et al., 2020), but the presence of core microbial elements also protects the host against fungal infections (Fraune et al., 2015).

*Hydra* polyps actively shape the composition of their associated bacterial community through the secretion of antimicrobial peptides (Fraune et al., 2010; Franzenburg et al., 2013; Augustin et al., 2017). As a result, laboratory maintained *Hydra* species differ in the bacterial community (Franzenburg et al., 2013), and show long-term association with the host, partly reflecting differences observed in their natural habitats (Fraune and Bosch, 2007). Furthermore, components of the microbiota are at least partly transmitted vertically to embryos through a process controlled by maternal antimicrobial peptides (Fraune et al., 2010; Minten-Lange and Fraune, 2020), providing an opportunity for coevolution between host physiology and microbial diversity. However, factors driving variation in the microbial composition and diversity of natural *Hydra* populations remained so far unexplained.

Here, we aimed to understand the factors that shape bacterial diversity in natural *Hydra* populations. To this end, we collected *Hydra* polyps belonging to three different co-existing species (*H. oligactis*, *H. vulgaris* and *H. circumcincta*) from 21 Central European locations. We asked whether bacterial diversity in *Hydra* is affected by (1) sampling population ID, (2) water body type, (3) nutrient load of sampling population, (4) host species, and (5) host reproductive state. In line with previous studies (e.g., Fraune and Bosch, 2007) we predicted that the three *Hydra* species will be associated with distinct microbial communities and were interested in finding out how consistent these species differences are across a range of distinct populations. Furthermore, we hypothesized that the type of habitat could influence the microbial diversity from which host-associated microbial communities are assembled and therefore recorded, for each location, whether it was standing or flowing water and categorized them in terms of nutrient load: meso-eutrophic, eutrophic or hyper-eutrophic. Finally, we hypothesized that life history stage of the host (specifically, whether it was reproducing sexually or asexually) can affect the diversity of microbial taxa associated with the host because of the altered physiology associated with sexual reproduction. In at least one of the three species (*H. oligactis*), sexual reproduction is associated with marked reductions in somatic maintenance functions, including loss of regeneration ability, stem cell depletion and disappearance of nematocytes (stinging cells) important for food capture (Sebestyén et al., 2018), and these physiological changes could also affect the ability to regulate host-associated microbes.

We found that the sampling site (population) has the strongest effect on α- and β-diversity, followed by the type of the water body, while the host factors (species and reproduction mode) had a much weaker, but consistent effect on the bacterial diversities. The results showed that environmental factors were most strongly associated with changes in the microbial community while the bacterial communities still specifically reflect the host species.

## Materials and Methods

### Field Sampling

Samples were collected from 21 water bodies (20 located in Hungary, 1 in Romania; Table 1), between 7th November and 27th November 2019. At each location we collected *Hydra* from multiple locations that were at least 2 m distance from each other.

**TABLE 1:** Location, sample size, type and trophic state of water bodies from which *Hydra* polyps were collected[1].

Population ID	Sample size	Coordinates	Type of water body	Category	Trophic state
M26	9	47.58746°N; 21.14895°E	River	Flowing	Hypereutrophic
M28	21	47.67119°N; 20.86334°E	Oxbow lake	Standing	Eutrophic
M31	7	47.04021°N; 18.06975°E	Lake	Standing	Eutrophic
M34	6	46.76484°N; 17.26994°E	Canal	Flowing	Eutrophic
M44	17	48.03405°N; 21.07803°E	Oxbow lake	Standing	Hypereutrophic
M67	10	46.76848°N; 18.61604°E	River	Flowing	Hypereutrophic
M70	8	46.77169°N; 17.63603°E	Creek	Flowing	Mesoeutrophic
M71	15	46.75138°N; 17.56861°E	Creek	Flowing	Eutrophic
M72	21	46.70316°N; 17.38126°E	Canal	Flowing	Hypereutrophic
M78	2	47.69665°N; 20.68656°E	Creek	Flowing	Mesoeutrophic
M79	18	47.69032°N; 20.74361°E	Canal	Flowing	Hypereutrophic
M83	15	48.17484°N; 21.61385°E	Oxbow lake	Standing	Eutrophic
M84	15	47.26047°N; 20.52008°E	Oxbow lake	Standing	Hypereutrophic
M85	15	47.17998°N; 20.31362°E	Oxbow lake	Standing	Hypereutrophic
M86	3	47.14387°N; 20.25956°E	Oxbow lake	Standing	Hypereutrophic
M89	28	46.85608°N; 19.99031°E	Oxbow lake	Standing	Hypereutrophic
M90	25	46.82147°N; 20.00077°E	Oxbow lake	Standing	Hypereutrophic
M107	10	48.17199°N; 21.50517°E	Oxbow lake	Standing	Eutrophic
M108	11	48.12438°N; 21.44716°E	Oxbow lake	Standing	Eutrophic
M109	10	47.68255°N; 20.82135°E	Oxbow lake	Standing	Hypereutrophic
R12	10	47.88977°N; 23.31119°E	River	Flowing	Mesoeutrophic

*Hydra* sampling sites were categorized as standing (lakes) or flowing (rivers, creeks and canals) water bodies. Furthermore, we also categorized trophic level of water bodies as mesoeutrophic, eutrophic or hypereutrophic based on personal observations of the presence of algal blooms and macrophyte cover during the vegetation period, proximity to agricultural areas and hence exposure to agricultural run-off and involvement of the water bodies in commercial fishing and angling activities. In the hypereutrophic category, there is usually a significant nutrient load (mostly nutrients from agricultural areas and nutrients from fish farming) which can result in frequent and significant algal blooms. In water bodies of the eutrophic category, algal blooms are common, but the nutrient load is not so high (mainly nutrients from fish farming and fishing). In waters belonging to the mesotrophic category, algal blooms are occasionally observed with lower nutrient load.

We located *Hydra* by placing small pieces of aquatic macrophytes into sterile plastic containers. Polyps found attached to these pieces of vegetation were gently removed with an automatic pipette and a sterile tip on the collection site, placed individually into sterile Eppendorf tubes and brought to the laboratory on the day of collection in a cool box.

In the laboratory, each polyp was visually inspected under a stereo microscope (Euromex Stereoblue), while still in the Eppendorf tube. We tentatively assigned species identity of *Hydra* polyps based on visual inspection of morphological characters (type and shape of gonads, tentacle length and appearance in asexual buds). We also categorized polyps into the following groups: asexual (having at least a bud), sexual male (with mature testes), sexual female (with mature ovaries), sexual immature (with a distinct yellow swelling on the body column but without clearly developed testes or ovaries) or non-reproductive (without buds or gonads; Sebestyén et al., 2018; Miklós et al., 2021).

DNA was extracted from whole polyps that were first gently washed with filtered lake water to remove debris attached to the polyps. Isolating DNA from whole polyps implies that for each *Hydra* individual we sampled bacteria located inside the gut cavity, on the outer surface of the polyps and those located within the tissue (all of which are known to host components of the microbiota). Furthermore, this also means that microbes only transiently associated with the host (e.g., originating from the food items ingested by *Hydra*, from the surrounding water or from the biofilms covering the macrophytes to which polyps attach) could also be detected.

After washing, polyps were frozen at −80°C and DNA extraction followed within 1 week of sample collection, using a chloroform/isoamyl alcohol extraction method [detailed description of extraction protocol described in supplementary of Miklós et al. (2021)]. From the extracts, 5 μl was used for 16S sequencing (see below for details). To further verify *Hydra* species identity, we also performed a PCR reaction on each of the samples with primers specific for *Hydra vulgaris* HSP70 (Steele et al., 1996). PCR reactions were performed at two temperatures: 56 and 64°C. This primer pair gives a clear signal on both temperatures for *H. vulgaris*, while no signal is detected in *H. circumcincta*. In *H. oligactis*, a clear PCR product is only detected at 56°C. The final species identity assigned to samples is the consensus of visual inspection and PCR results.

### 16S rRNA Gene Amplicon Sequencing

The 16S rRNA gene was amplified using uniquely bar-coded primers flanking the V1 and V2 hypervariable region (27F–338R) with fused MiSeq adapters and heterogeneity spacers in a 25-μl PCR (Fadrosh et al., 2014). For the traditional one-step PCR protocol, we used 4 μl of each forward and reverse primer (0.28 μM), 0.5 μl dNTPs (200 μM each), 0.25 μl Phusion Hot Start II High-Fidelity DNA Polymerase (0.5 Us), 5 μl of HF buffer (Thermo Fisher Scientific, Inc., Waltham, MA, United States), and 1 μl of undiluted DNA. PCRs were conducted with the following cycling conditions [98°C, 30 s; 30 × (98°C, 9 s; 55°C, 60 s; 72°C, 90 s); 72°C, 10 min; 10°C, infinity] and checked on a 1.5% agarose gel. The concentration of the amplicons was estimated using a GelDoc™XR + System coupled with Image Lab™Software (BioRad, Hercules, CA, United States) with 3 μl of O’GeneRulerTM100 bp Plus DNA Ladder (Thermo Fisher Scientific, Inc., Waltham, MA, United States) as the internal standard for band intensity measurement. The samples of individual gels were pooled into approximately equimolar sub-pools as indicated by band intensity and measured with the Qubit dsDNA br Assay Kit (Life Technologies GmbH, Darmstadt, Germany). Sub-pools were mixed in an equimolar fashion and stored at −20°C until sequencing.

Library preparation for shotgun sequencing was performed using the NexteraXT kit (Illumina) for fragmentation and multiplexing of input DNA following the manufacturer’s instructions. Amplicon sequencing was performed on the Illumina MiSeq platform with v3 chemistry (2 × 300 cycle kit), while shotgun sequencing was performed on an Illumina NextSeq 500 platform *via* 2 bp × 150 bp Mid Output Kit at the IKMB Sequencing Center (CAU Kiel, Germany).

### Data Analysis

The initial analysis 16S-sequencing reads was performed in the Qiime2 framework v2020.8.0 (Bolyen et al., 2019). Adapter trimming was done using Qiime2’s cutadapt (Martin, 2011) with following adapters: fwd: AGRGTTYGATYMTGGCTCAG, rev: TGCTGCCTCCCGTAGGAGT. Surviving reads were required to have a minimum length of 20 bp, all other settings were left to defaults. For denoising we employed the Qiime2 DADA2 plugin (Callahan et al., 2016) using following settings: “–p-trunc-len-f 290 –p-trunc-len-r 235 –p-trunc-q 10 –p-trim-left-f 9 –p-trim-left-r 9.” The resulting representative sequences were used to annotate bacterial taxonomy by using the feature-classifier classify-consensus-blast tool of qiime2 (Camacho et al., 2009) against the SILVA132 database (Yilmaz et al., 2014) using default settings. Furthermore, phylogenetic relationships between ESVs were inferred using the mafft and fasttree implementation (Katoh, 2002; Price et al., 2009) of Qiime2 using default settings. After trimming, denoising, phylogenetic and taxonomic annotation we exported the results and used the R statistical programming language v4.1.0 to analyze and plot the data (R Core Team, 2021).

Samples were filtered for sequencing depth and samples with less than 3,368 read counts were removed (14 in total). The threshold is the result of different filtering steps we have taken. First we filtered for rare ESVs, by removing all ESVs which contributed less than 1% overall sequencing reads per sample and were not found in at least 3 samples in total. We then used a data driven threshold to remove the samples we considered outlier by calculating the logarithm of the read-counts per sample and scaling the data. Checking the distribution of these transformed and scaled read-counts revealed a rather heavy tail on the left hand side, so we decided to symmetrize the data by removing all samples which were smaller than −1 × max(scaled(log(read-counts))). Afterward we excluded again all ESVs which had no reads in the remaining samples. After that we checked for rarefraction curves in the low count samples and detected no major issues with sampling, but were uneasy with samples which remained in the data set and had read-counts as low as 888 in total. We thus decided to remove the lower 5% of the samples, resulting in a threshold of exactly 3,368 reads. This procedure resulted in a total of 1,864 ESVs (from 11,290) and a total of 94.86% of the initial sequencing reads remaining in the data set. 16S sequences and annotation can be found in Supplementary Tables 1, 2.

For general data handling and plotting we used the data.table v1.14.0, ggplot2 v3.3.5 and cowplot packages, respectively (Wickham, 2016; Wilke, 2020; Dowle and Srinivasan, 2021). For β-diversity calculations we normalized and variance stabilization transformed the raw read count data using the vst() function of the DESeq2 package v.1.32.0 (Love et al., 2014), while α-diversity was calculated directly on the raw read count matrix. To avoid infinity values by log-transformation during data normalization we added a single read count to every field in the read count matrix. Since variance stabilizing transformation results in negative entries of the read count matrix, we shifted the whole matrix to only positive values by addition of −1 × min(v) where v is the vector of the flattened matrix. α- and β-diversity measures were calculated using the functionality of the phyloseq and vegan packages (McMurdie and Holmes, 2013; Oksanen et al., 2020). After testing for several measures for α- and β-diversity (data not shown) we obtained best results with the Shannon index for α-diversity and Bray–Curtis dissimilarity for β-diversity and used these measures throughout the study. UMAP ordination were calculated on the β-diversity measures using the umap() function of the umap v0.2.7.0 package (Konopka, 2020), employing the dist-option, while PCA calculation where performed on the same data using the base package prcomp() function, employing scaling and centering. Since we did not rarefy the read counts prior to analysis of alpha diversity, we tested the difference of rarefraction analysis compared to raw species count (Chao1) and Shannon index calculation (Supplementary Figure 1) by calculating rarefied samples using the functionality of the vegan package. Basic statistical analyses were performed with the R base package (linear models, Kruskal–Wallis tests) while PERMANOVA on the β-diversity was performed using the adonis2() function implemented in the vegan package. For the PERMANOVA tests, we regarded population (PopID) as random factor within the permutation block design and the method for testing was the “Terms”-option—equivalent to type I ANOVA tests on main effects. Since type I tests for ANOVA analysis are sensitive to order of appearance in the design formula, we tested several models switching each factor to the last position of the formula to check its significant association to the data.

Linear mixed models and statistical testing was performed using the lme4 v1.1.27.1, lmerTest 3.1.3, car v3.0.11 and lsmeans v2.30.0 packages (Bates et al., 2015; Lenth, 2016; Kuznetsova et al., 2017; Fox and Weisberg, 2019). All models were checked for normal distribution of error terms and random factor coefficients as well as for improved data explanation compared to the null-model. Furthermore, all models use all tested factors as explanatory variables and the population (PopID) as random effect.

Differentially abundant species were calculated using the DESeq2 package v.1.32.0 with default settings and following the design formula: “∼reproduction mode + species + nutrient load + water body type.” Bacteria were regarded as differential abundant when the adjusted *p*-value was smaller 0.05 and the fold change of read counts was at least twofold (absolute log2 fold change > 1). DESeq2 was chosen for calculation of indicator species for twofold reasons: On the one hand recent comparative studies suggests that DESeq2 is suitable for handling different sample sizes much better than rarifying or fraction based analysis (McMurdie and Holmes, 2014) while being more sensitive with good error control rate if compared to other common methods (Nearing et al., 2021). On the other hand, we tested several methods (LefSe, Ancom2, Simmer) with our data set and found DESeq2 to be the only one sensitive and performant enough to report reasonable results.

For all analyses, *p*-value adjustments were performed by the method of false discovery rate correction (Benjamini and Hochberg, 1995).

Relative abundance per condition is the fraction of sample weighted read counts summed by members of each bacterial order. This results in contributions from 0–1 where each sample contributes equally to the fraction calculated.

## Results

### Microbial Diversity in Three Different *Hydra* Species in the Field

**FIGURE 1:**
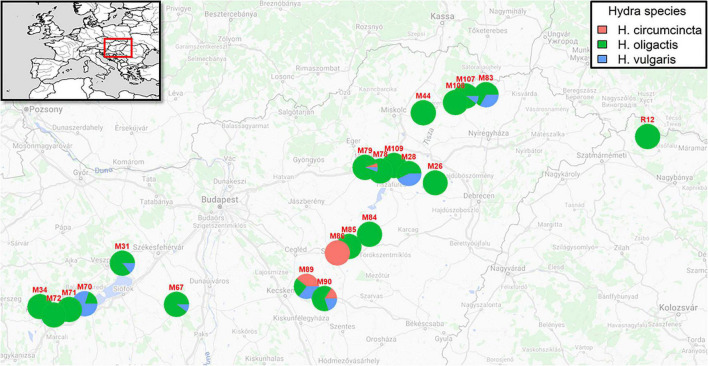
Map showing sampling locations and proportion of *Hydra* species. The inset shows the geographic location of the sampling area in the Europe map. The pie charts represent the proportion of species detected in each water body.

In 2019, we sampled 265 (251 after quality filtering) *Hydra* polyps from 21 different locations in Hungary and Romania (Figure 1 and Table 1). Within this sampling effort, we took samples from 12 oxbow lakes, 1 lake, 3 rivers, 3 creeks and 3 canals (Table 1) within a geographic range of about 460 km (Figure 1). Overall, we identified polyps from three different coexisting species: *Hydra oligactis*, *Hydra vulgaris* and *Hydra circumcincta*. While *Hydra oligactis* could be detected in 20 water bodies, *Hydra vulgaris* was found in nine and *Hydra circumcincta* in four locations. Independent of the trophic state of the water body in almost all sampled habitats, *Hydra oligactis* was the dominant species. Only in two lakes (M89 and M90) we detected an even presence of all three species.

**FIGURE 2:**
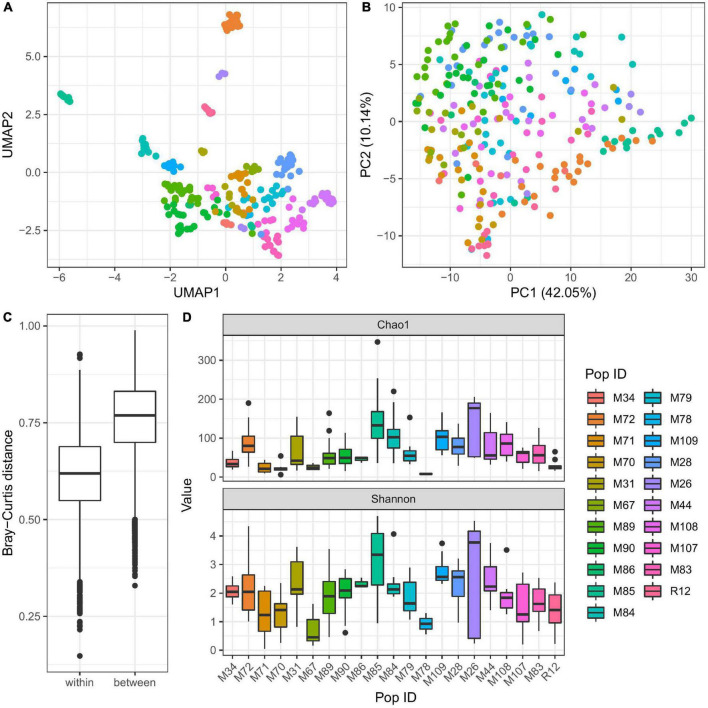
Influence of population identity on bacterial colonization in *Hydra*. UMAP dimensionality reduction **(A)** and PC-analysis **(B)** of β-diversity (Bray–Curtis) shows separation of the samples by population, thus environmental conditions. The separation of microbial communities by population is supported by Adonis test (*R*^2^ = 0.41, *p* ≤ 0.001 with 999 permutations, Table 2). Bray–Curtis **(C)** dissimilarities within one population are generally smaller than those between populations. Furthermore, population ID is a major determinant for alpha diversity **(D)** (linear model on Shannon index, *p* ≤ 0.001, Table 3).

To evaluate the factors contributing to the diversity of bacterial colonization in *Hydra*, we extracted DNA from all sampled *Hydra* polyps and compared their associated microbiota by 16S rRNA sequencing. PC and UMAP clustering analyses revealed that the microbial diversity of *Hydra* polyps sampled is mainly influenced by population identity (Figures 2A,B and Table 2). This was also reflected in Bray–Curtis dissimilarities where polyps from different water bodies show significantly larger dissimilarities as polyps from the same water body (Figure 2C).

**TABLE 2:** Statistical analysis of host and environmental factors influencing bacterial diversity of *Hydra*.

	Univariate PERMANOVA			Multivariate PERMANOVA		
Condition	*R* ^2^	*F*	*p* value	*R* ^2^	F	*p* value
Pop ID	0.41	7.99	<0.001	N/A	N/A	N/A
Water body type	0.04	10.64	<0.001	0.03	9.04	0.0001
Trophic state	0.05	7.05	<0.001	0.04	6.16	0.2570
Species	0.04	5.46	<0.001	0.04	5.84	0.0001
Reproduction	0.01	1.78	0.0012	0.01	1.72	0.2848

In addition to the differences in beta-diversity, alpha-diversity of the bacterial communities associated with *Hydra* polyps was also significantly affected by the population identity (Figure 2D and Supplementary Table 3). Interestingly, samples from the water bodies showing the most distinct UMAP clustering (M26, M72, M85) harbored the highest bacterial diversity estimated by chao 1 and Shannon index (Figure 2D).

### Environmental Effects on Bacterial Diversity in Three Different *Hydra* Species

Given that population identity was the most important factor explaining differences in bacterial diversity, we analyzed the contribution of the two environmental factors “water body type” and “trophic state” of the different water bodies to explain bacterial diversity associated with *Hydra* polyps (Table 1).

**FIGURE 3:**
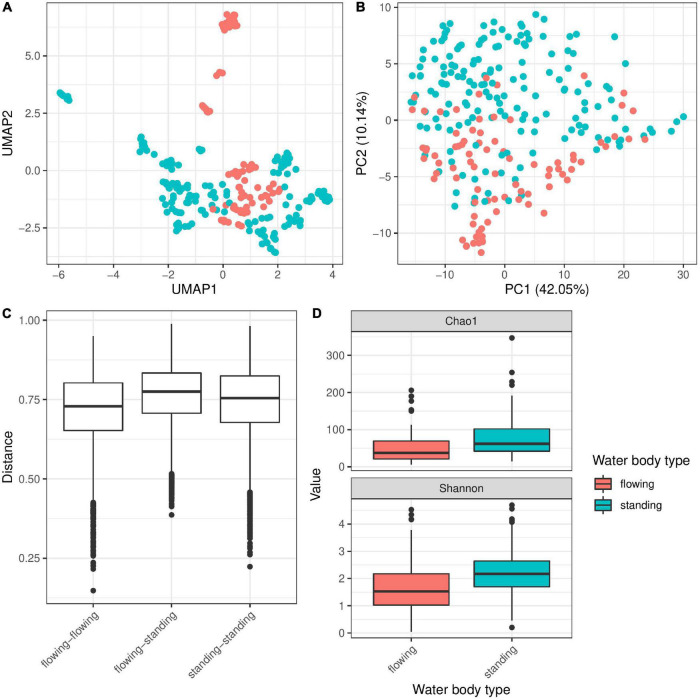
Influence of water body type on bacterial colonization in *Hydra*. UMAP dimensionality reduction **(A)** and more clearly PC-analysis **(B)** of β-diversity (Bray–Curtis) show separation of the samples by water body type. This separation of microbial communities is supported by PERMANOVA test (*R*^2^ = 0.04, *p* ≤ 0.001 and *R*^2^ = 0.03, *p* ≤ 0.001 with 999 permutations each for univariate and multivariate PERMANOVA). Bray–Curtis **(C)** dissimilarities are smallest between flowing water bodies, while dissimilarities between standing only and flowing and standing water bodies are higher. In support of this result, α-diversity is significantly smaller in flowing water than in standing water bodies for α-diversity measures chao 1 and Shannon **(D)** (univariate linear model: *p* ≤ 0.001, multivariate linear mixed model on Shannon index with population ID as random effect *p* ≤ 0.001).

The 21 different water bodies were clustered into two different categories—flowing (rivers, creeks and canals) and standing (lakes and oxbow lakes; Table 1). Testing the contribution of this factor to bacterial diversity associated with *Hydra* revealed a significant association (Figure 3 and Table 2) explaining around 4% of bacterial variation. Interestingly, Bray–Curtis dissimilarities were smallest between flowing water bodies, while dissimilarities between standing only and flowing and standing water bodies were higher (Figure 3C). This indicates that *Hydra*’s microbiota was more constrained in diversity in flowing water bodies as those in standing water bodies. In support of this result, polyps living in running water, like river and creek, harbor a significantly lower bacterial α-diversity, than polyps living in standing water (Figure 3D). Bacterial taxa that were more frequently present on *Hydra* polyps in flowing water belong to Sphingobacteriaceae, Myxobacteria and Pseudomonadales, while Betaproteobacteria were more dominant on *Hydra* polyps living in standing water (Supplementary Figure 2).

**FIGURE 4:**
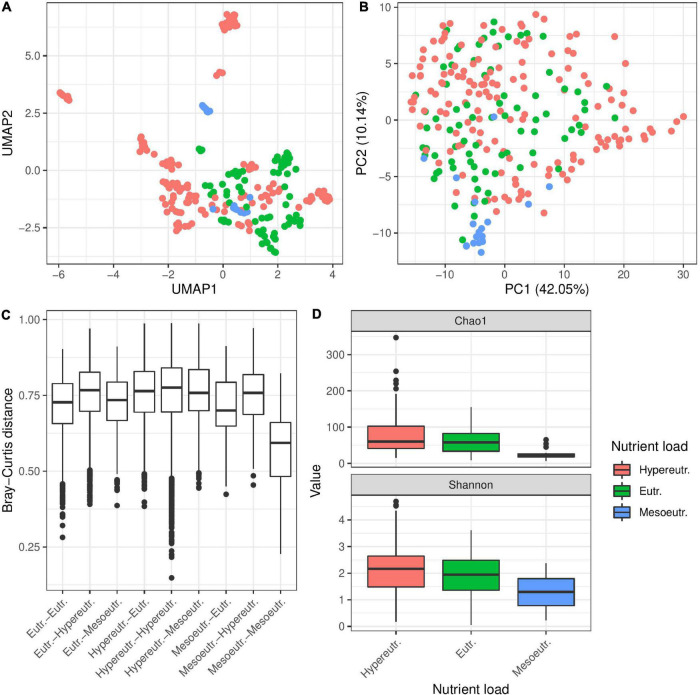
Influence of trophic state of water body on bacterial colonization in *Hydra*. UMAP plots **(A)** and PC ordination **(B)** of β-diversity (Bray–Curtis) show separation of the samples by trophic state of the water body. Separation of microbial communities by trophic state is supported by univariate PERMANOVA (*R*^2^ = 0.05, *p* ≤ 0.001 with 999 permutations, Table 2) but not in multivariate PERMANOVA (*R*^2^ = 0.04, *p* = 0.2570, with 999 permutations, PopID as random effect, all other factors as cofactor). Bray–Curtis dissimilarities **(C)** are smallest between mesoeutrophic water bodies, while dissimilarities between all other pairs of trophic states are higher. This indicates that *Hydra*’s microbiota is more constrained in diversity in mesoeutrophic water bodies as those in standing water bodies. In support of this result, α-diversity is significantly smaller in mesoeutrophic water bodies than in eutrophic and hypereutrophic water bodies **(D)** in univariate linear models (*R*^2^ = 0.070, *p* ≤ 0.2E-4, on Shannon index). However, multivariate models do not support the influence of trophic state on bacterial diversity (linear mixed model on Shannon index with population as random effect, all other factors as cofactor, X ^2^ = 4.385, *p* = 0.112).

In addition to habitat type, we categorized the different sampling sites according to their trophic state into mesoeutrophic, eutrophic and hypereutrophic waters (Table 1 and Figure 4). Comparing bacterial diversity of *Hydra* polyps collected from environments with different trophic levels revealed a significant clustering, if the factor was tested individually (Table 2 and Figures 4A,B). In particular, *Hydra* associated bacterial communities from mesoeutrophic water bodies were significantly less diverse than bacterial communities associated with *Hydra* polyps living in eu- or hypereutrophic water bodies (Figure 4D and Supplementary Figures 3A,B). This difference was also evidenced by the fact that bacterial communities from *Hydra* polyps living in mesotrophic habitat were more similar to each other than to bacterial communities from polyps living in eu- or hypertrophic habitats (Figure 4C and Supplementary Figure 3). The indicator analysis revealed that bacteria belonging to Campylobacterales and Cytophagales were specifically enriched on polyps living in mesoeutrophic habitats (Supplementary Figure 3C). However, the statistical difference was lost if testing the trophic level in context of the multivariate models (Tables 2, 3), which indicates that the nutritional effect had only minor contribution to bacterial diversity. The explanatory power for bacterial diversity is probably encoded within the water body type (flowing or standing) where we detected highest correlations between factors (Supplementary Figure 6).

**TABLE 3:** Statistical analysis of biotic and abiotic factors determining diversity of the associated bacteria of *Hydra*.

	Univariate linear model			Multivariate linear mixed model	
Condition	*R* ^2^	*F*	*p* value	X ^2^	*p* value
Pop ID	0.343	6.013	5.4E-12	N/A	N/A
Water body	0.085	23.205	6.3E-6	7.148	0.008
Trophic state	0.070	9.355	0.2E-4	4.385	0.112
Species	0.021	2.673	0.089	19.014	7.4E-5
Reproduction	0.001	0.172	0.842	9.892	0.007

### Host Effects on Bacterial Colonization in *Hydra*

**FIGURE 5:**
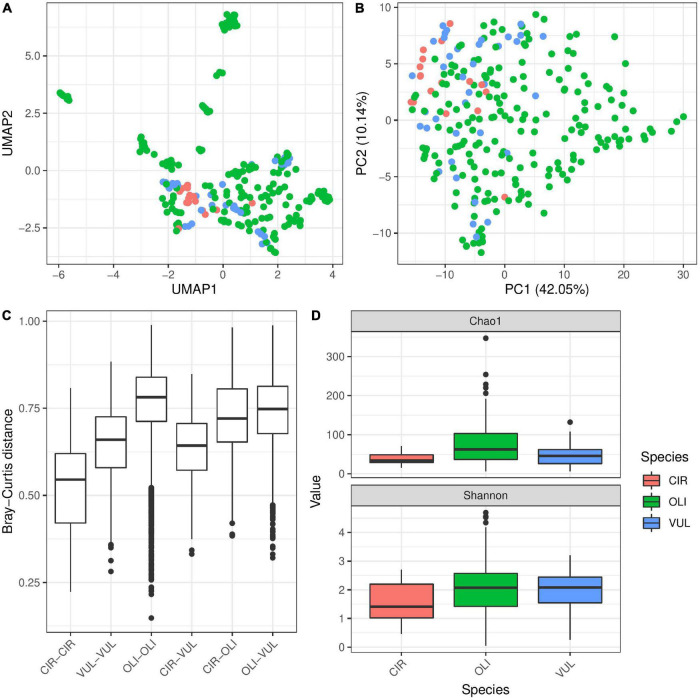
The influence of host species on microbial diversity in *Hydra*. UMAP **(A)** and PCA **(B)** ordination plots for the Bray–Curtis dissimilarity show moderate clustering of samples by species. The clustering is supported by testing for differences in Bray–Curtis dissimilarity by PERMANOVA (*R*^2^ = 0.04, *p* ≤ 0.001 with 999 permutations, univariate and multivariate models, Table 2). The boxplot for Bray–Curtis dissimilarity **(C)** shows reduced values for β-diversity within *H. circumcincta* samples compared to the rest, while *H. oligactis* seems to have largest dissimilarity values within one species, indicating that *H. oligactis* is able to host a more diverse microbial community than the other species. The dissimilarity between *H. circumcincta* and *H. vulgaris* is generally smaller than between the other species pairs indicating more similar microbial communities between these two species. α-diversity **(D)** is similarly affected by the host species (*p* = 0.089 and *p* ≤ 0.001 for univariate linear model and multivariate linear mixed model, respectively Table 3). *H. circumcincta* showed the lowest α-diversity, which was also significantly different compared to the other species (Supplementary Table 3).

In addition to environmental factors, host species identity also explained 4% bacterial diversity variation associated with the different *Hydra* polyps (Table 2). While bacterial communities associated with *Hydra oligactis* revealed only weak clustering, the bacterial diversity associated with *H. circumcincta* was highly distinct (Figures 5A,B and Supplementary Figures 4A,B). This was also demonstrated by the fact that within Bray–Curtis distances of bacterial communities associated with *H. circumcincta* were smallest, followed by *H. vulgaris* (Figure 5C). The within distances in bacterial communities associated with *H. oligactis* showed the highest distances, indicating a less constrained diversity of bacterial associations. A similar trend was evident from the higher alpha-diversity in *H. oligactis* compared to the microbiota of *H. circumcincta* and *H. vulgaris* (Figure 5D) which was also statistically supported if we correct the analysis for environmental cofactors (Table 3). Similar effects could be observed by analyzing samples from a single population separately to test the effect in environmentally similar settings. We performed PERMANOVA analysis for species effect in population M89 and M90 and found strong association of β-diversity with it, explaining up to 23% variability (Supplementary Figures 7, 8 and Supplementary Table 3). Additionally, we observed a similar trend with *H. oligactis* being associated with higher Shannon-indices (Supplementary Figures 7, 8), but found statistical significance only in population M90 (Supplementary Table 4). Bacterial taxa that colonized *H. oligactis* with higher frequency belonged to the Bacteroidia, while *H. circumcincta* was associated more frequently with Rickettsiales (Supplementary Figure 3). Interestingly, most bacteria that were specifically associated with one of the three species (indicator species) belonged to the Betaproteobacteria (Supplementary Figure 3).

**FIGURE 6:**
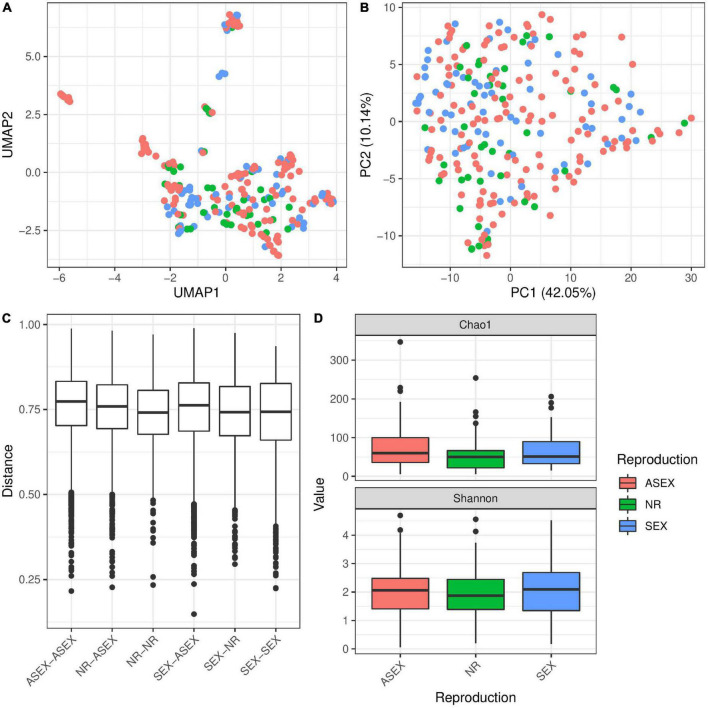
The influence of reproduction mode on microbial diversity in *Hydra*. UMAP **(A)** and PCA **(B)** ordination plots for the Bray–Curtis dissimilarity show no clustering of samples by reproduction mode. However, univariate PERMANOVA retrieved a significant effect of reproduction mode on β-diversity (*R*^2^ = 0.01, *p* = 0.0012, 999 permutations, Table 2). However, multivariate PERMANOVA results do not support a statistical effect in differences due to the mode of reproduction in Bray–Curtis dissimilarity (*R*^2^ = 0.01, *p* = 0.2848, 999 permutations, Table 2). Similarly, the boxplot for Bray–Curtis dissimilarity **(C)** shows no major difference between the different pairs of reproduction modes. However, α-diversity **(D)** is affected by the reproduction mode in a multivariate linear mixed model (*p* = 0.007, Table 3), an effect which is not supported by univariate linear models (*p* = 0.842, Table 3).

In addition, we tested the reproduction status of the polyps and if it is influencing the microbial diversity. Reproducing animals were categorized depending on the presence of gonads (sexual), presence of reproductive buds (asexual) or absence of both (non-reproductive). In general we can say that there were only minor effects of the reproduction state which were reflected in the microbial community. Neither UMAP nor PCA clustering by reproductive mode were clearly visible (Figures 6A,B) and Bray–Curtis distances tended to be slightly smaller within non-reproductive animals as compared to all other pairs (Figure 6C). This small effect could be detected in the univariate PERMANOVA model (*p* = 0.0012, Table 2) while it was not recovered in the multivariate PERMANOVA (*p* = 0.2848, Table 2) indicating that correlations with other factors were important to explain the small differences in distances (e.g., species assignment, Supplementary Figure 6). Similarly, we observed only small effects of reproduction mode on α-diversity (Figure 6D)—where we found no support in the univariate linear model on the Shannon index (*p* = 0.842, Table 3). Interestingly, there was a detectable difference in the α-diversity which was assigned to reproduction in the multivariate linear mixed model (*p* = 0.007, Table 3), which was driven by a difference between sexual and asexual animals (Supplementary Table 3). Testing for differences in α- and β-diversity in single populations (M89 and M90) showed no significant association between reproduction and diversity measures (Supplementary Figures 7, 8 and Supplementary Tables 3, 4), except in univariate PERMANOVA in population M89. This difference was mainly driven by species differences which showed perfect co-linearity between *H. circumcincta* and sexual reproduction in this case (Supplementary Figure 9 and Supplementary Table 3). The bacterial composition between reproduction modes were very similar, with noticeable differences in the expansion Betaproteobacteria in non-reproductive animals, while Rickettsiales were associated in slightly higher abundance in sexual polyps, compared to the other conditions (Supplementary Figures 5A,B). Accordingly, we identified only a few indicator species, most of them belonging to the Betaproteobacteria (Supplementary Figure 5C).

### Abundance of Curvibacter in Field Sampled *Hydra* Polyps

**FIGURE 7:**
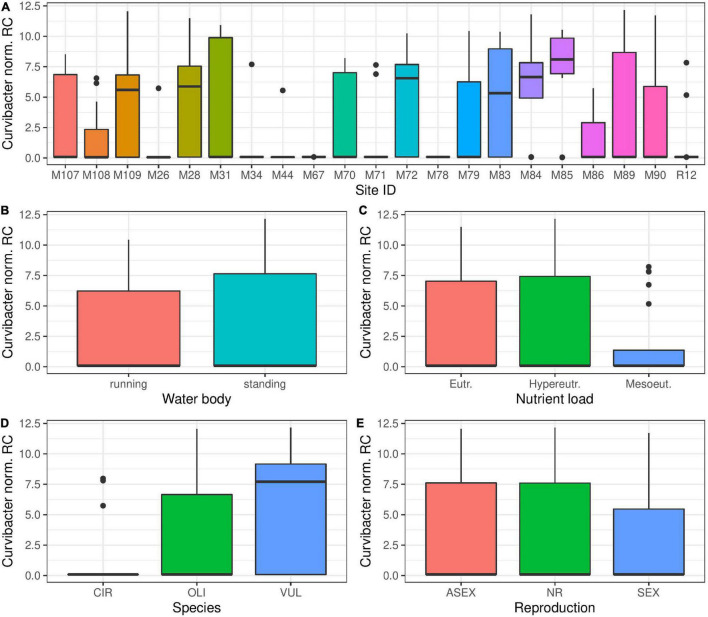
*Curvibacter*, the main colonizer in laboratory strains of *Hydra* is consistently found in wild *Hydra* samples. The presence of *Curvibacter* species is dependent on the environmental conditions of different sampling sites **(A)** (Kruskal–Wallis, *p* < 0.001). However, nutrient load of the water **(B)** and type of water body **(C)** have no significant effect on the amount of colonizing *Curvibacter* (Kruskal–Wallis, *p* > 0.05, respectively). *H. vulgaris* polyps show a higher prevalence of Curvibacter colonization **(D)** (Kruskal–Wallis, *p* < 0.001). The mode of reproduction seems to have a small effect on the amount of *Curvibacter* colonization **(E)** (Kruskal–Wallis, *p* < 0.002). CIR, *H. circumcincta*; OLI, *H. oligactis*; VUL, *H. vulgaris*; ASEX, asexual reproduction; NR, non-reproductive; SEX, sexual reproduction.

*Curvibacter* bacteria are one of the most prevalent bacterial groups colonizing *Hydra* polyps in the laboratory (Franzenburg et al., 2013). While *Curvibacter* could be detected in most *Hydra* populations sampled, (Figure 7A) the abundance of this group of bacteria was much lower than in laboratory maintained polyps. Interestingly, the presence of *Curvibacter* species varies strongly within the different *Hydra* populations (Figure 7A), but not for the tested environmental factors: water body type and nutrient load (Figures 7B,C). The overall *Curvibacter* distribution to different *Hydra* species was consistent with results from laboratory experiments (Franzenburg et al., 2013), where *H. vulgaris* showed a higher prevalence of *Curvibacter* colonization (Figure 7D). The mode of reproduction seemed to have a small effect on the amount of *Curvibacter* colonization, with sexually reproducing polyps having lower amounts of *Curvibacter* in their microbial community (Figure 7E).

## Discussion

Here, we investigated factors associated with changes in taxonomic diversity of host-associated microbes in three species of freshwater *Hydra* (*Hydra oligactis*, *H. vulgaris* and *H. circumcincta*) coexisting in Central European water bodies. We found that: Firstly, *Hydra* populations differed markedly in bacterial composition with population identity being the most important predictor of microbial α- and β-diversity. Secondly, the type of habitat explained differences in bacterial diversity. Thirdly, we detected significant differences in bacterial diversity among host species (least diverse in *H. circumcincta*). Fourthly, we did not find a strong effect of reproductive mode (sexual vs. asexual) associated with bacterial composition. We discuss these findings in turn below.

Source population appeared the most important factor explaining diversity of the community of microbes associated with *Hydra* hosts. This observation is somewhat surprising given that the geographical scope of the sampling was not very large (∼460 km between the most distant populations) and sampling locations were mostly similar habitat types (lowland freshwater bodies). Furthermore, *Hydra* is known to host distinct and highly specific microbe species (Fraune and Bosch, 2007; Franzenburg et al., 2013; this study) that are partly vertically transmitted from parent to offspring (Fraune et al., 2010), which might suggest that microbial composition is inherited across generations rather than being assembled from the environment. Nonetheless, microbial communities, characteristic for the different habitats seem to transiently colonize *Hydra* which eventually result in long-term associations with bacteria that are either added to (or replace) host-specific microbes. For instance, if functionally similar but taxonomically different microbes are present in distinct habitats, then *Hydra* individuals might end up with taxonomically different, but functionally very similar microbial communities. Population-specific variation in microbial composition similar to our results has been described in aquatic vertebrates and explained as the result of variation in interrelated environmental conditions such as temperature, geography, water quality and chemistry affecting the composition of local microbial communities and ultimately, host-associated microbes (reviewed in: Sehnal et al., 2021).

Additionally, population effects on microbial diversity might be further enhanced through genetic differentiation of the host (e.g., due to limited gene flow between populations). If host populations are isolated, then their ancestral microbial composition might also become differentiated due to stochastic losses and gains in microbial taxa or adaptive differences in host genetics and immunity (Chaston et al., 2016; Glasl et al., 2019; Frankel-Bricker et al., 2020). In this case, population differences in microbial composition and diversity would reflect the evolutionary history and population structure of the host. Although we cannot, at present, fully exclude this possibility, a previous study of the population genetics of *H. oligactis* detected very limited spatial genetic structuring among some of the populations that were also sampled in this study (Miklós et al., 2021). Therefore, we consider it unlikely that population structure or differences in host genotype could explain the site-dependence of microbial diversity.

We also have to note that some of the diversity of host-associated microbes detected in this study might stem from taxa that are only transiently associated with the host, e.g., if locally present bacterial taxa (found either in the water, the substrates to which polyps attach or the food they consume) settle on the surface or basal disk of polyps, or accumulate in their gastric cavity. This possibility could be investigated in the future through laboratory studies, e.g., by maintaining polyps sampled from different locations under similar conditions (same culture medium and food) and testing whether the differences in their microbial community persist under identical environmental conditions. However, such transiently associated taxa cannot explain the species differences observed in this study, since polyps belonging to distinct species were often collected from the same population (also see below). Furthermore, while transiently associated microbial taxa are often discounted as unimportant and most studies focus on the so-called “core” microbial community, recent research shows that such transiently associated microbial species can strongly affect the resident community (Amor et al., 2020). Therefore, we think that the population differences in microbial diversity observed by us could have important functional consequences for the host, although this idea needs to be tested experimentally in the future.

Given that sampling site identity had such an important effect on microbial diversity, what could be the driving force behind these differences? Comparing microbial diversity of *Hydra* polyps collected from distinct habitat types we found that trophic level and water body type (i.e., standing vs. flowing) significantly affected microbial diversity in single-predictor models, such that *Hydra* originating from flowing water and/or with reduced nutrient load had reduced microbial diversity. This suggests that the physical properties of the habitat or the higher nutrient content of eutrophic/hypertrophic water bodies could alter the diversity of host-associated microbes, e.g., through influencing the number of bacterial taxa and the diversity of their metabolic function that are present in these habitats and can colonize animals living therein (Dickerson and Williams, 2014; Kiersztyn et al., 2019). Similar observations have been previously made under experimentally altered nutrient loads e.g., in corals (Jessen et al., 2013; Shaver et al., 2017). However, we have to mention that clearly discerning the role of distinct habitat features in driving microbial diversity is difficult based on our data, because the two categorizations (trophic level on one hand and standing vs. flowing on the other) correlated with each other, with complete separation of factor levels in some cases (e.g., all mesoeutrophic habitats were in the flowing category). Therefore, future studies will require a more balanced sampling of distinct habitat types to clearly ascertain which feature of the habitat affects microbial diversity. On another note, it is noteworthy that the explained variability in β-diversity for water body type and trophic state is low (4% and 3%, respectively) compared to the variability which can be assigned to the population (41%), which indicates that other environmental factors contribute to the assemblage of the microbiota in *Hydra* than those we have assessed. Given the fact that trophic levels of the water body has a large impact on free living bacterial diversity (Dickerson and Williams, 2014; Kiersztyn et al., 2019), it seems to be less important for the *Hydra* associated microbiota. There could be habitat specific *Bdellovibrio* and like organisms (BALOs) which explain part of the changes in diversity. BALOs have been described as predatory bacteria feeding on other gram-negative bacteria (Sockett, 2009) and they have been associated with an increase in bacterial diversity if present in the microbial community of different metazoans (Johnke et al., 2020). Differences in the abundance and species prevalence of BALOs in the tested habitats might thus contribute to the diversity differences observed.

We also found that microbial diversity was significantly influenced by host species, even after controlling for host population, implying that interspecific differences are persistent. Polyps belonging to the three distinct species included in our study often co-occurred, sometimes physically very close to each other, on the same pieces of substrate. Therefore, their distinct microbiota is most likely the result of species-specific host-microbe associations. Similar consistent patterns of association between specific microbial taxa and hosts have been described in a number of animal groups from sponges to mammals (Yildirim et al., 2010; Lee et al., 2011; Carlos et al., 2013; Brooks et al., 2016; Youngblut et al., 2019), and are often evident in coexisting species of aquatic animals (Sehnal et al., 2021). Species-specific microbial composition has also been described previously for *Hydra* (Fraune and Bosch, 2007; Franzenburg et al., 2013). Our observations strengthen those of Fraune and Bosch (2007) on a larger sample size and wider geographical scope, involving multiple *Hydra* populations.

Although significant differences between host species were detected, interspecific variation was relatively low (lower than interpopulation variation). Of the three species, the smallest microbial diversity was observed in *H. circumcincta* and the largest in *H. oligactis*. Moreover, we found that *H. circumcincta* had a more consistent microbial composition compared to the two other species. This species is phylogenetically the most distinct of the three (Schwentner and Bosch, 2015), and is also biologically different from the rest (e.g., it is a simultaneous hermaphrodite; Reisa, 1973), offering potential explanations for their distinct microbiota. The observation of relatively low interspecific variation echoes a recent study which found limited differentiation among the microbiota of taxonomically diverse co-existing freshwater zooplankton (Eckert et al., 2021). Interestingly, while we found clearly differentiated microbiota of the three *Hydra* species in some populations, no such differentiation was apparent in others, suggesting that the interaction between host species and sampling site might explain microbial diversity in *Hydra*. Since *H. oligactis* was found to be the most widespread species in this study, its higher microbial diversity could be the result of exposure to more diverse environments. Alternatively, the opposite might be also true, such that a more diverse microbiota confers the host greater adaptability and colonization of more diverse habitats (Voolstra and Ziegler, 2020). Again we should note that the effect of species on α- and β-diversity is small compared to the effect of populations. This lets us conclude that species specific differences are very robust, but are implemented in an environmental dependent context—which after all is the main predictor of microbial diversity in *Hydra* associated bacteria. However, even if the species differences were small, they might still be functionally important. For instance, in laboratory experiments with *Hydra*, single bacterial taxa can provide important fitness benefits to the host (Fraune et al., 2015; Taubenheim et al., 2020).

Compared to sampling site and host species identity, the reproductive status of *Hydra* polyps appeared to be less important in determining microbial diversity. We expected a significant difference between sexual and asexual polyps because sexual individuals have markedly different physiology compared to asexual ones. We detected this difference in α-diversity in a multivariate model considering all other covariables, but the signal was very weak, indicating that mode of reproduction has only a marginal effect on the microbial community. In *H. oligactis*, sexual reproduction is associated with reduced regeneration ability, depletion of somatic stem cells, nematocytes involved in food capture and nerve cells and increased mortality risk (Yoshida et al., 2006; Sebestyén et al., 2018; Tomczyk et al., 2019, 2020; Ngo et al., 2021). Nerve cells in *Hydra* are prominently involved in the production of antimicrobial peptides that shape microbial composition (Augustin et al., 2017), therefore their loss could be expected to impact microbial composition. Furthermore, sexually reproducing *Hydra* with developing eggs show markedly increased expression of the antimicrobial peptide periculin, which shapes microbial composition in the developing embryo while it is still attached to the parent animal (Fraune et al., 2010). Finally, a previous study detected consistent differences between sexual and asexual individuals of the snail *Potamopyrgus antipodarum* (Takacs-Vesbach et al., 2016). Yet, we found only little difference in the microbial diversity of sexual and asexual *Hydra* polyps, indicating that the physiological changes described above have only limited effects on overall microbial diversity. On the other hand, it is also possible that sexual individuals collected by us were in early stages of sexual development (since we sampled in the first part of the sexual period) and physiological differences were not advanced enough to generate a difference in microbial composition.

To summarize, we found, using a large dataset of field-collected *Hydra* polyps, that variation in the diversity of host-associated microbes is mainly associated with the local environment, with eutrophication potentially playing a role in increasing microbial diversity. Additionally, the microbial composition changed significantly with host species in *Hydra*, with *H. circumcincta* displaying the least diverse and most consistent microbial communities. In comparison, host reproductive mode did not explain changes in microbial diversity. The mechanisms through which the local environment interacts with host species/genotype in assembling the microbiota remains an interesting topic for further investigations in this system.

## Data Availability Statement

The original contributions presented in the study are included in the article/Supplementary Material, further inquiries can be directed to the corresponding authors. Short reads were submitted to the SRA repository under project number: PRJNA795254. Code to analyze data is available at: https://github.com/Porthmeus/GeoBacPhylo.

## Author Contributions

JTö, SF, and JTa designed the research. MM and JTö performed the field sampling and DNA extraction. JTa analyzed the data. All authors wrote the manuscript.

## Conflict of Interest

The authors declare that the research was conducted in the absence of any commercial or financial relationships that could be construed as a potential conflict of interest.

## Publisher’s Note

All claims expressed in this article are solely those of the authors and do not necessarily represent those of their affiliated organizations, or those of the publisher, the editors and the reviewers. Any product that may be evaluated in this article, or claim that may be made by its manufacturer, is not guaranteed or endorsed by the publisher.

## Supplementary Material

Supplementary Figure 1 Rarefraction analysis shows independence of species richness of sequencing depth. **(A)** Rarefraction plots for each sample and population shows that most bacterial diversity is sampled with fewer reads per sample, indicating that sampling depth is deep enough to guarantee independence of bacterial diversity and sequencing depth. To test whether the chosen measures for α-diversity are indeed independent of sampling depth, we calculated species richness on rarefractions (subsampling of reads in each sample to the number of reads of the smallest sample). **(B)** shows the distribution of rarefaction values across the different sampling populations (compare to Figure 2D). Furthermore we correlated these to the Shannon **(C)** and chao1 **(D)** indices calculated on the uncorrected samples—both correlations are high (*R*^2^ = 0.8 and 0.97, respectively) and highly significant (*p* < 0.001 for both).

Supplementary Figure 2 Most indicator species for flowing and standing water bodies belong to the Bacteroidetes or Proteobacteria. Comparing overall relative abundance (mean relative abundance of all samples) of bacteria in flowing and standing water bodies shows drastic changes in microbial composition of *Hydra* **(A)**. In flowing waters we observed an increase in Pseudomonadales, Myxococcales and Sphingobacteriales, while standing water samples were enriched in uncultured Gracilibacteria, Holosporales, and Betaproteobacteria, as indicated by boxplots of relative abundance per sample **(B)**. Most of the indicator species on the species level (ESV) for both flowing and standing water bodies belong to the phylum of Proteobacteria and Bacteroidetes **(C)**. Certain Leptospiraceae species are indicative for running waters only in our study, while some Xanthomonadales, Rickettsiales, Chitinophagaceae and Cytophagaceae species are specific for standing waters.

Supplementary Figure 3 There are only minor differences in the bacterial abundances between different trophic states of the water bodies. The changes of the overall relative bacterial abundance between the different trophic states are only minor, while the mesoeutrophic water bodies are more different than the other two states **(A)**. The largest difference can be observed in the reduction of Chitinophagaceae, Gracilibacteria and Holosporales in mesoeutrophic waters, while Cytophagaceae are relatively increased **(B)**. Accordingly, mesoeutrophic waters can be associated with more indicator species (ESV level) than the other two trophic states **(C)**.

Supplementary Figure 4 Different *Hydra* species are colonized by distinct microbial communities. The microbiota of all *Hydra* species are dominated by Betaproteobacteria which comprise >50% of the bacteria **(A)**. At the same time microbial communities of *H. circumcincta* are more distinct from the other two species. The largest difference can be observed in the expansion of Rickettsiales in *H. circumcincta* **(B)**. Accordingly, *H. circumcincta* can be associated with more indicator species (ESV level) than the other two trophic states **(C)**.

Supplementary Figure 5 Non-reproductive polyps are distinct in microbial composition. The overall bacterial composition between different reproductive states is very similar, but largest changes can be observed for non-reproductive polyps **(A)**. This change is driven by the expansion of Betaproteobacteria in non-reproductive polyps, to the expense of all other phyla **(B)**. Due to the overall similarity of the microbial communities there are only very few indicator species which could be identified for the reproductive state **(C)**.

Supplementary Figure 6 Correlation matrix for the tested environmental and biotic factors in multivariate statistics. To estimate the co-dependencies of the tested variables we calculated the Kendall correlation coefficient for each integer encoded **(A)** and hot-one encoded **(B)** level of all factors of interest. Absolute correlations coefficients never exceeded 0.5 outside one factor, indicating relatively uncorrelated data at hand. The data is derived from single polyp meta data associations, which would include pseudoreplication for the environmental factors, removing pseudoreplication results in following correlation coefficients: flowing—Eutr.: −0.2, flowing—Hypereutr.: −0.14, flowing—Mesoeutr.: 0.47 (opposite sign against standing water body).

Supplementary Figure 7 Analysis of individual population M89 recapitulates global patterns for species associated changes in the microbiota and no influence of reproduction on microbial changes. The different species form distinct clusters in UMAP representations **(A)** and PCA representation **(B)** of β-diversity, which finds statistical support in PERMANOVA analysis (*p* < 0.001 in multivariate and univariate PERMANOVA respectively, Supplementary Table 3). Lower Bray–Curtis distances can be observed within *H. circumcincta*, *H. vulgaris*, and *H. circumcincta*—*H. vulgaris* pairs, while all other pairs display higher diversity changes **(C)**—notably also within *H. oligactis* pairs higher dissimilarity indices are present. There are smaller Bray–Curtis distances in sexual reproducing animals **(D)**, which find support in univariate PERMANOVA (*p* = 0.004, Supplementary Table 3) but not in multivariate PERMANOVA (*p* = 0.59, Supplementary Table 3)—this divergence is caused by perfect collinearity of sexual reproduction and *H. circumcincta* thus the difference is caused by the species effect on β-diversity. α-diversity changes with different species **(E)** and mode of reproduction **(F)**, but finds no statistical support in linear models (*p* = 0.084, *p* = 0.093 for the univariate and multivariate linear model and species factor, *p* = 0.083 and *p* = 0.904 for univariate and multivariate model in reproduction, Supplementary Table 4).

Supplementary Figure 8 Analysis of individual population M90 recapitulates global patterns for species associated changes in the microbiota and no influence of reproduction on microbial changes. The different species form distinct clusters in UMAP representations **(A)** of β-diversity, while the effect is less clear in the PCA representation **(B)** which finds support in statistical analysis (*p* < 0.001 in multivariate and univariate PERMANOVA respectively, Supplementary Table 3). Lower Bray–Curtis distances can be observed within *H. circumcincta* and *H. vulgaris*, while all other pairs display higher diversity changes **(C)**—notably also within *H. oligactis* pairs higher dissimilarity indices are present. There are no obvious differences in the Bray–Curtis distances for the different reproduction categories **(D)**, which is also reflected in the statistical test for influence of reproduction mode on microbial diversity (*p* = 0.323 and *p* = 0.306 in univariate and multivariate PERMANOVA, Supplementary Table 3). α-diversity changes significantly with different species [**(E)** *p* = 0.002 for the univariate and multivariate linear model, respectively, Supplementary Table 4] but do not display significant differences with changes in reproductive mode [**(F)** *p* = 1 and *p* = 0.181 for univariate and multivariate linear model, Supplementary Table 4].
