# Supplementary figures and images for: Population Differences and Host Species Predict Variation in the Diversity of Host-Associated Microbes in Hydra
Source: Front Microbiol. 2022 Mar 3;13:799333. doi: 10.3389/fmicb.2022.799333 (PMC8927533; doi:10.3389/fmicb.2022.799333)

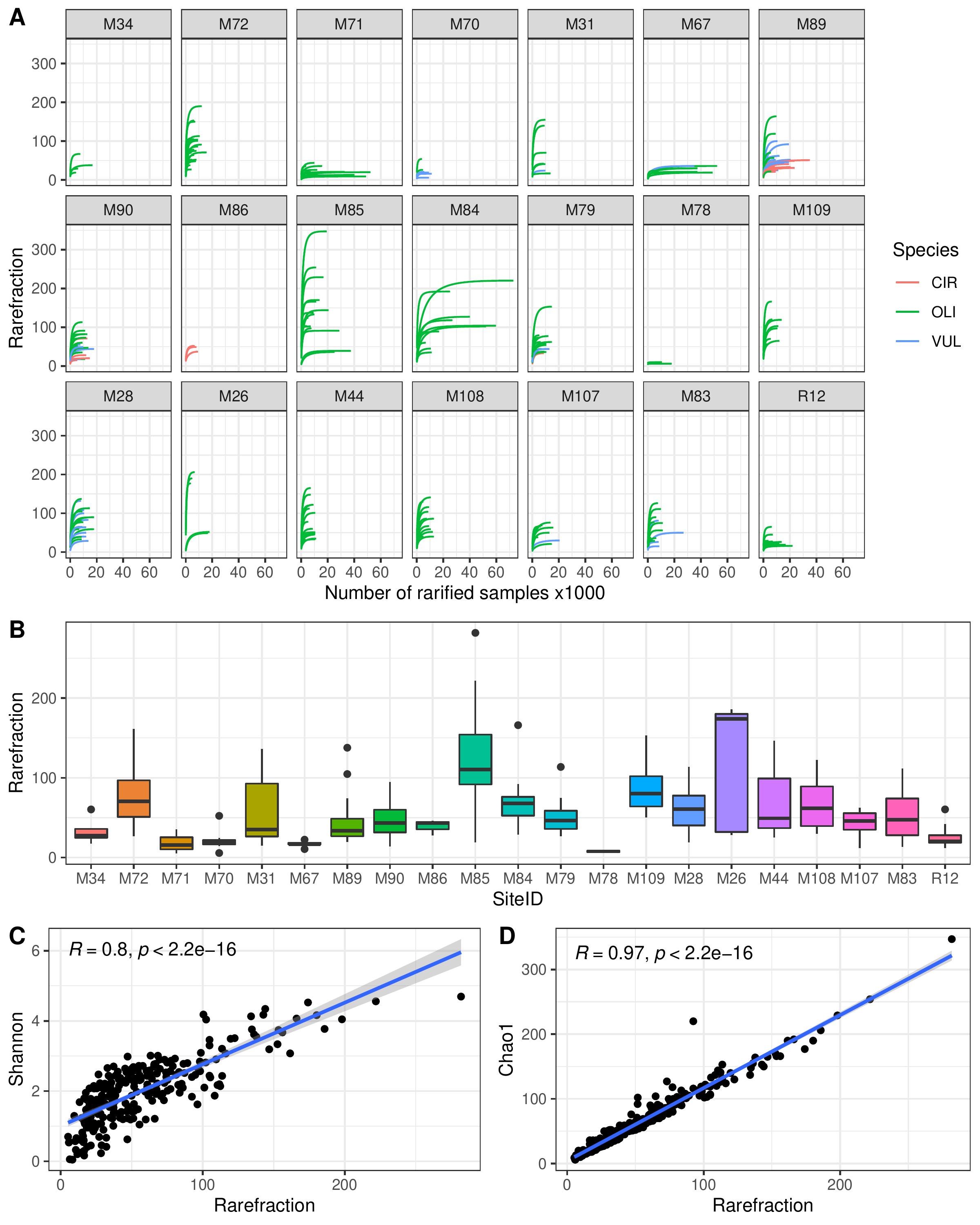

Supplement: Supplementary Figure 1 — Rarefraction analysis shows independence of species richness of sequencing depth. (A) Rarefraction plots for each sample and population shows that most bacterial diversity is sampled with fewer reads per sample, indicating that sampling depth is deep enough to guarantee independence of bacterial diversity and sequencing depth. To test whether the chosen measures for α-diversity are indeed independent of sampling depth, we calculated species richness on rarefractions (subsampling of reads in each sample to the number of reads of the smallest sample). (B) shows the distribution of rarefaction values across the different sampling populations (compare to Figure 2D). Furthermore we correlated these to the Shannon (C) and chao1 (D) indices calculated on the uncorrected samples—both correlations are high (R2 = 0.8 and 0.97, respectively) and highly significant (p < 0.001 for both). [file Image_1.JPEG]

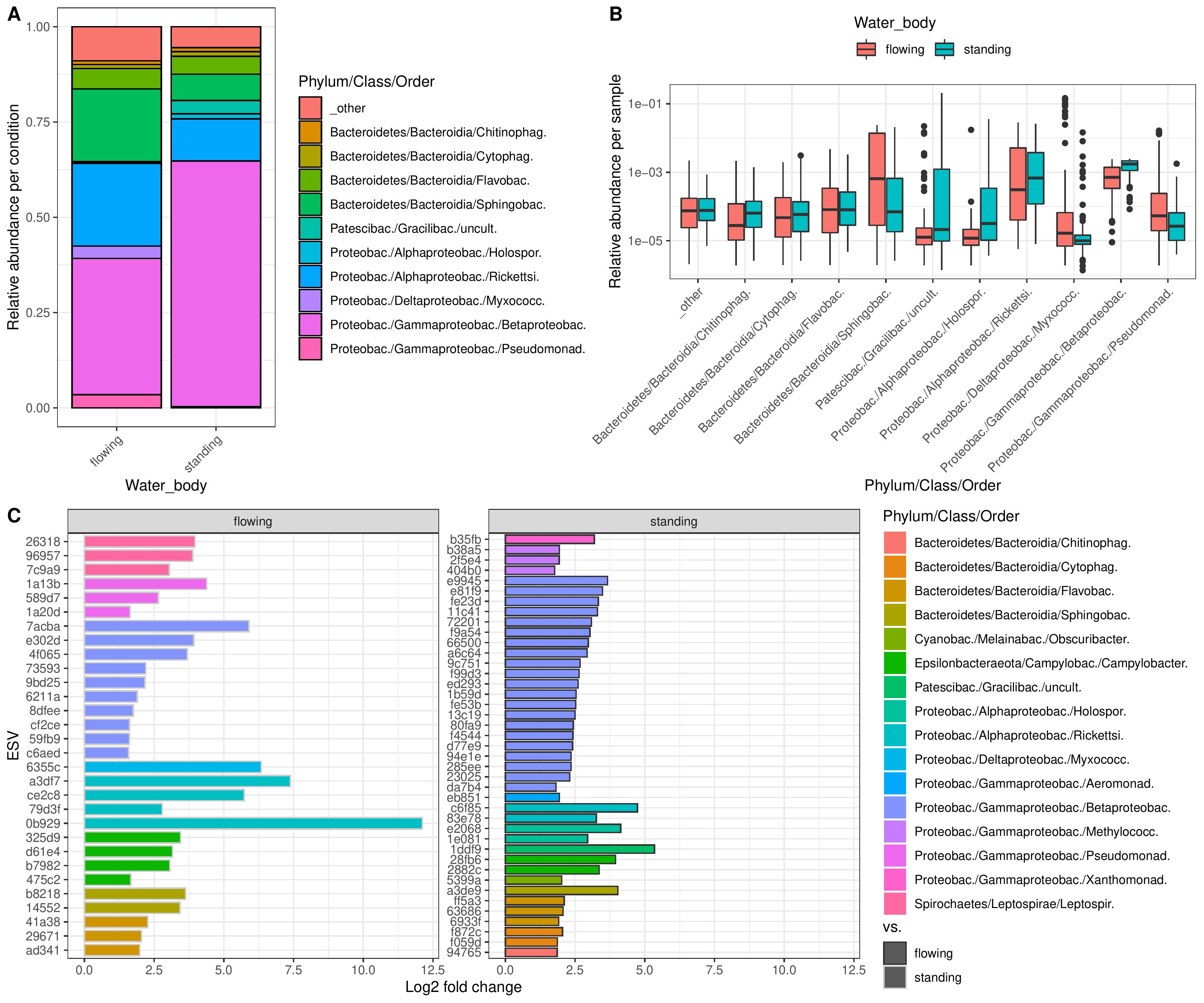

Supplement: Supplementary Figure 2 — Most indicator species for flowing and standing water bodies belong to the Bacteroidetes or Proteobacteria. Comparing overall relative abundance (mean relative abundance of all samples) of bacteria in flowing and standing water bodies shows drastic changes in microbial composition of Hydra (A). In flowing waters we observed an increase in Pseudomonadales, Myxococcales and Sphingobacteriales, while standing water samples were enriched in uncultured Gracilibacteria, Holosporales, and Betaproteobacteria, as indicated by boxplots of relative abundance per sample (B). Most of the indicator species on the species level (ESV) for both flowing and standing water bodies belong to the phylum of Proteobacteria and Bacteroidetes (C). Certain Leptospiraceae species are indicative for running waters only in our study, while some Xanthomonadales, Rickettsiales, Chitinophagaceae and Cytophagaceae species are specific for standing waters. [file Image_2.JPEG]

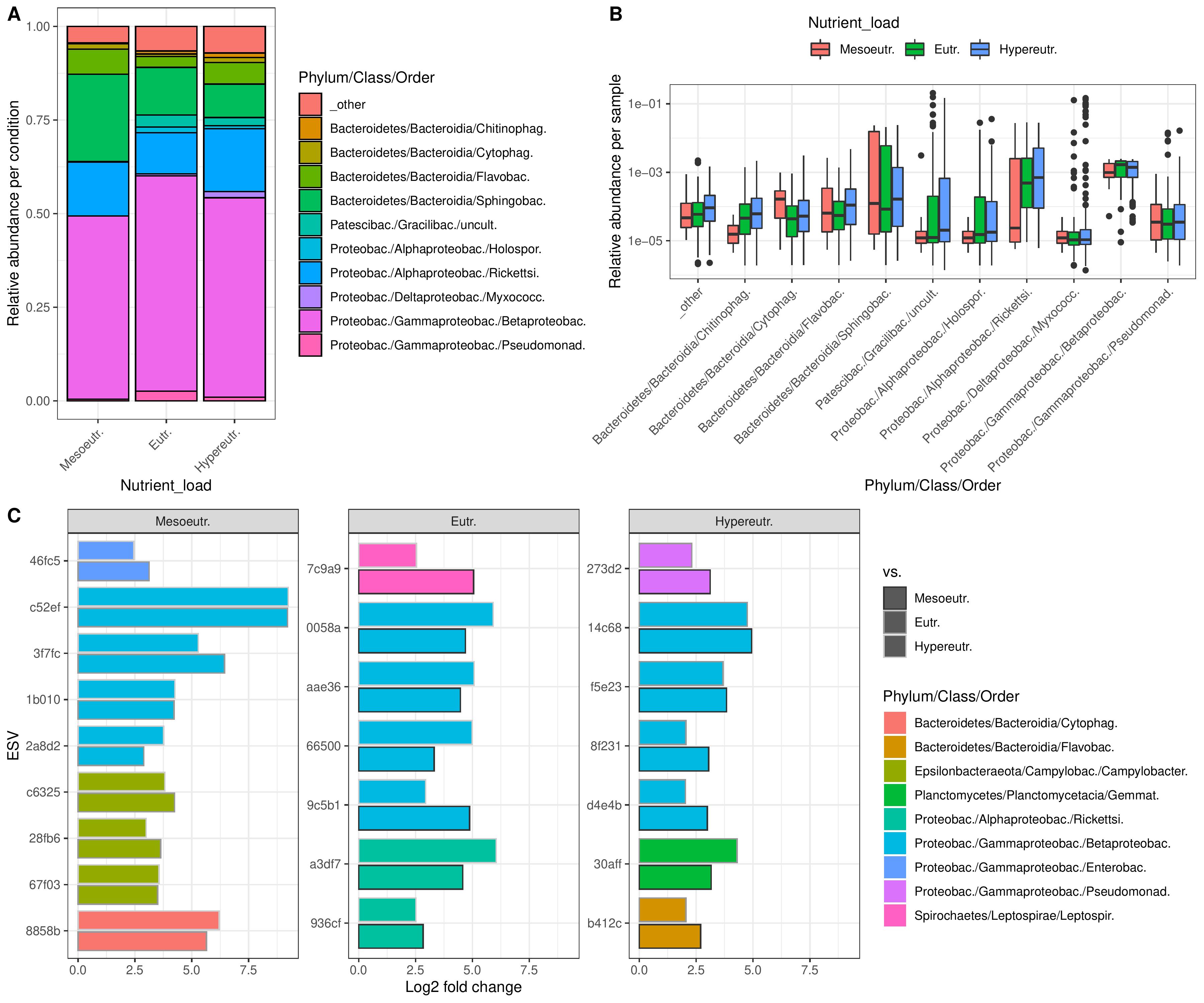

Supplement: Supplementary Figure 3 — There are only minor differences in the bacterial abundances between different trophic states of the water bodies. The changes of the overall relative bacterial abundance between the different trophic states are only minor, while the mesoeutrophic water bodies are more different than the other two states (A). The largest difference can be observed in the reduction of Chitinophagaceae, Gracilibacteria and Holosporales in mesoeutrophic waters, while Cytophagaceae are relatively increased (B). Accordingly, mesoeutrophic waters can be associated with more indicator species (ESV level) than the other two trophic states (C). [file Image_3.JPEG]

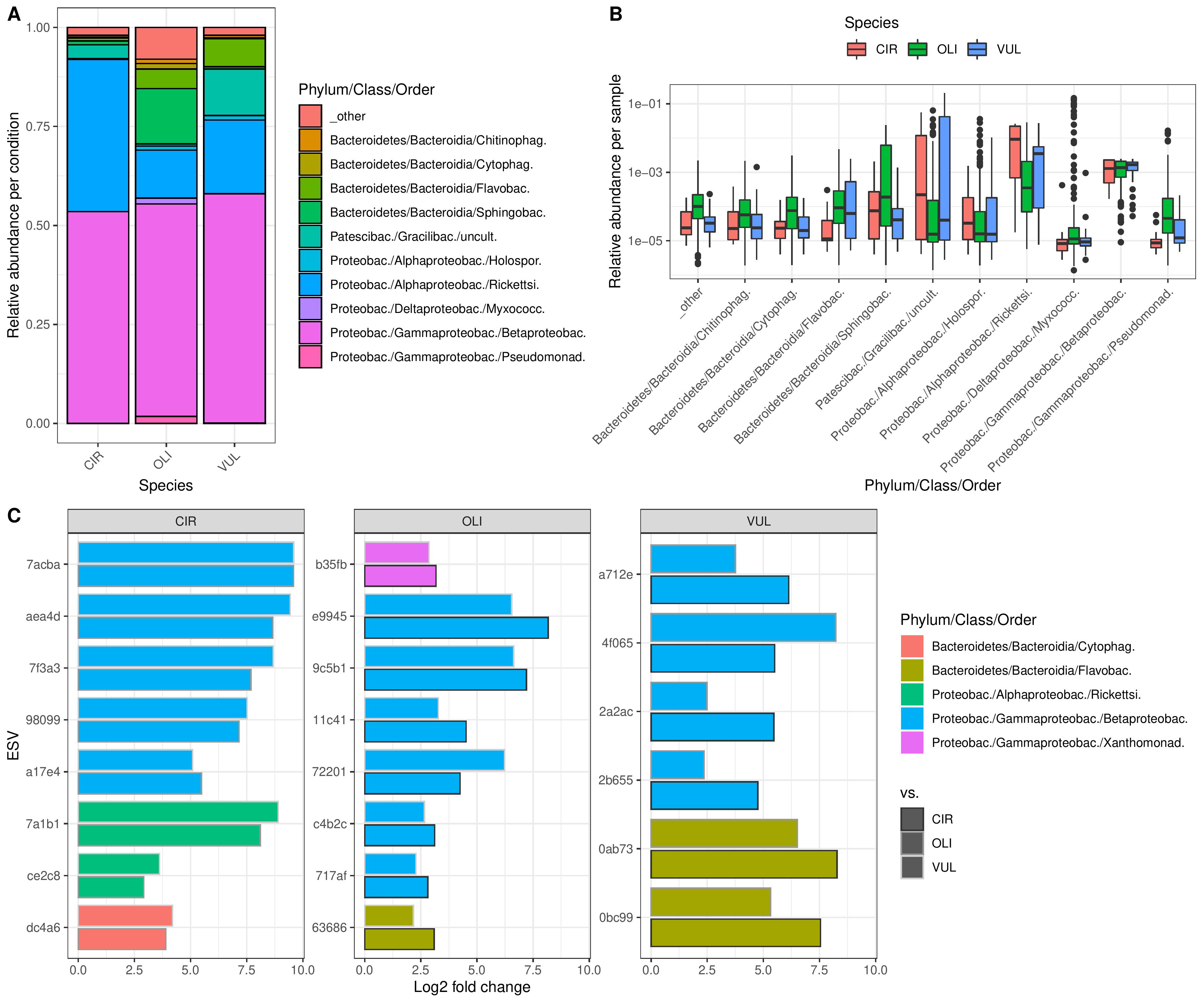

Supplement: Supplementary Figure 4 — Different Hydra species are colonized by distinct microbial communities. The microbiota of all Hydra species are dominated by Betaproteobacteria which comprise >50% of the bacteria (A). At the same time microbial communities of H. circumcincta are more distinct from the other two species. The largest difference can be observed in the expansion of Rickettsiales in H. circumcincta (B). Accordingly, H. circumcincta can be associated with more indicator species (ESV level) than the other two trophic states (C). [file Image_4.JPEG]

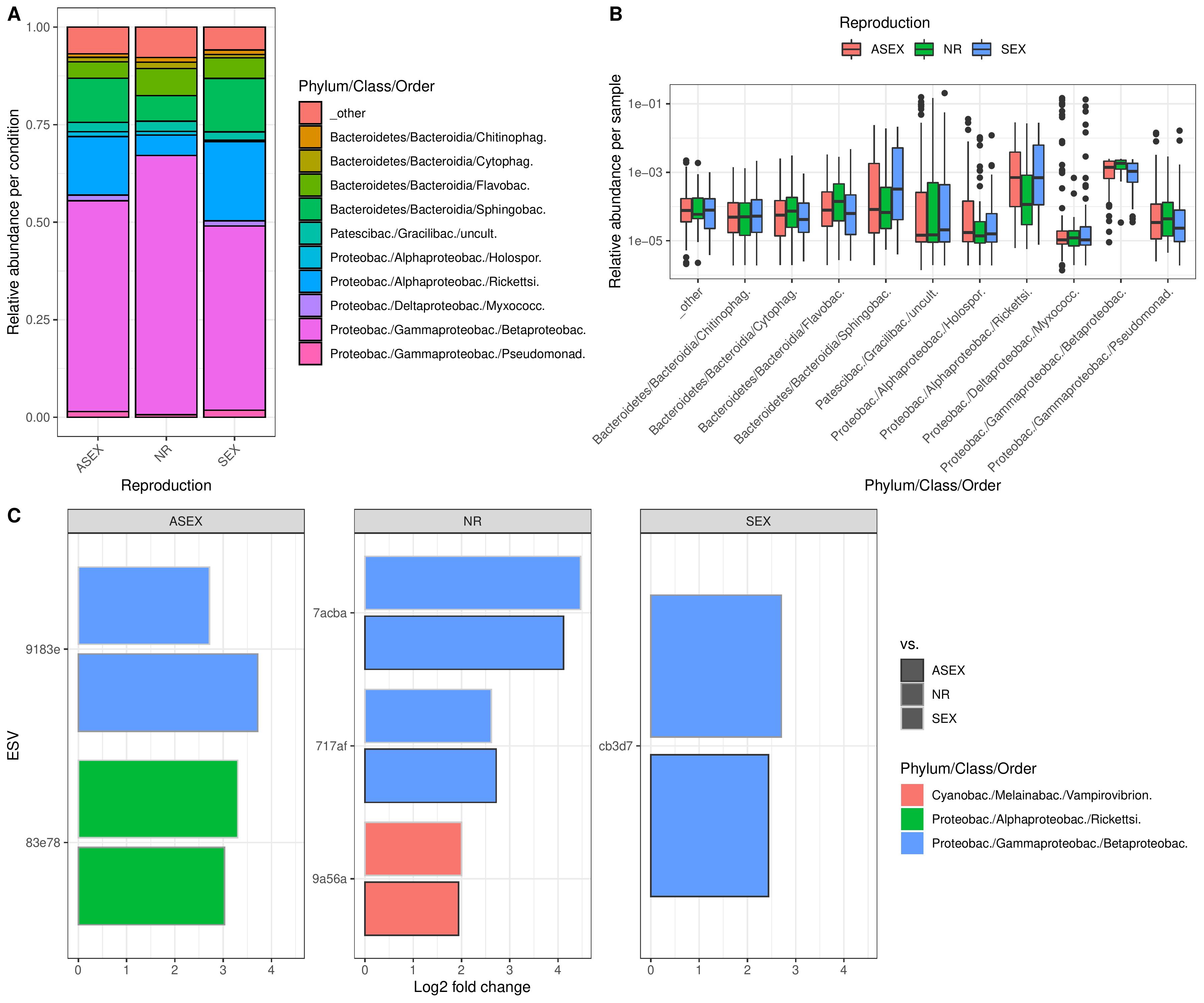

Supplement: Supplementary Figure 5 — Non-reproductive polyps are distinct in microbial composition. The overall bacterial composition between different reproductive states is very similar, but largest changes can be observed for non-reproductive polyps (A). This change is driven by the expansion of Betaproteobacteria in non-reproductive polyps, to the expense of all other phyla (B). Due to the overall similarity of the microbial communities there are only very few indicator species which could be identified for the reproductive state (C). [file Image_5.JPEG]

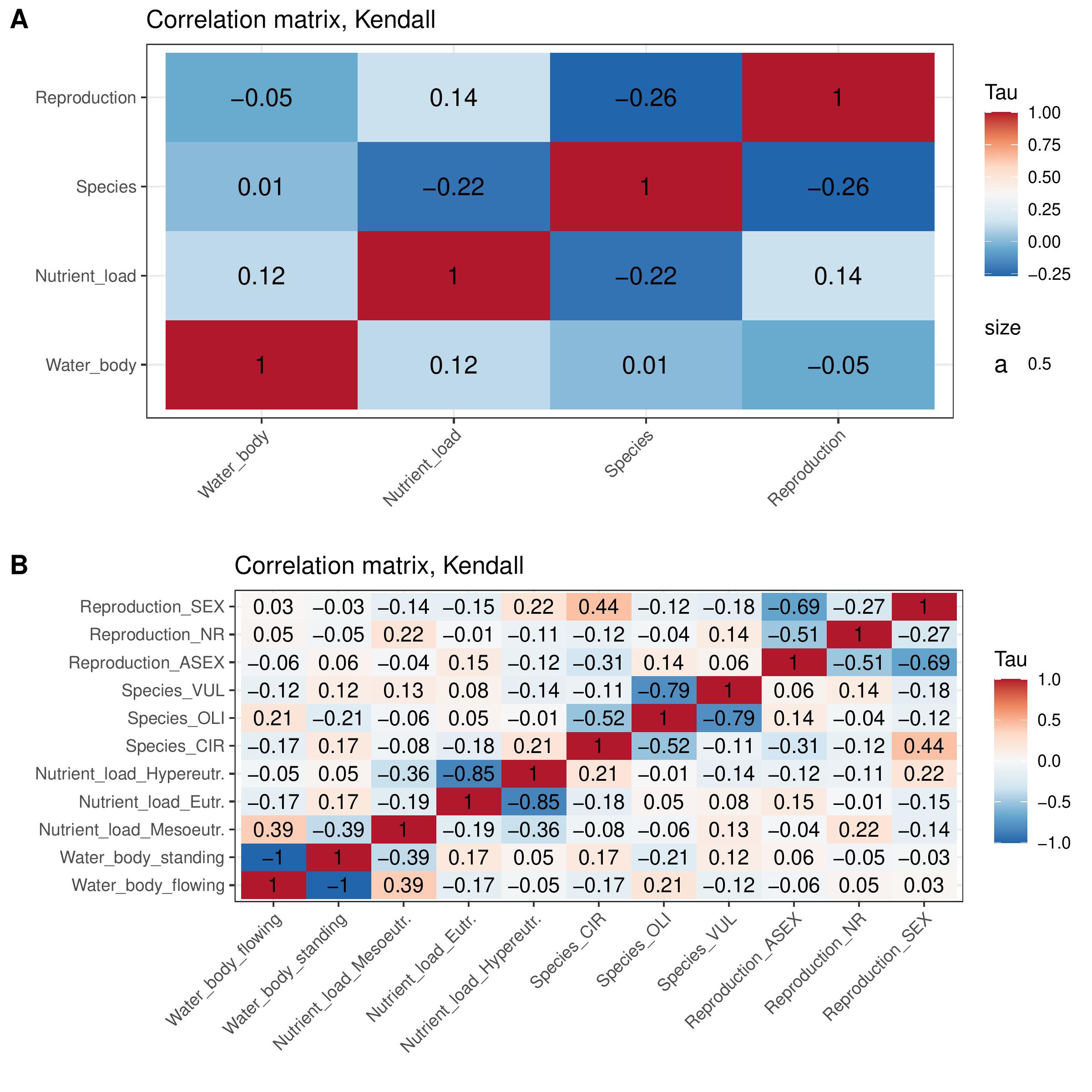

Supplement: Supplementary Figure 6 — Correlation matrix for the tested environmental and biotic factors in multivariate statistics. To estimate the co-dependencies of the tested variables we calculated the Kendall correlation coefficient for each integer encoded (A) and hot-one encoded (B) level of all factors of interest. Absolute correlations coefficients never exceeded 0.5 outside one factor, indicating relatively uncorrelated data at hand. The data is derived from single polyp meta data associations, which would include pseudoreplication for the environmental factors, removing pseudoreplication results in following correlation coefficients: flowing—Eutr.: −0.2, flowing—Hypereutr.: −0.14, flowing—Mesoeutr.: 0.47 (opposite sign against standing water body). [file Image_6.JPEG]

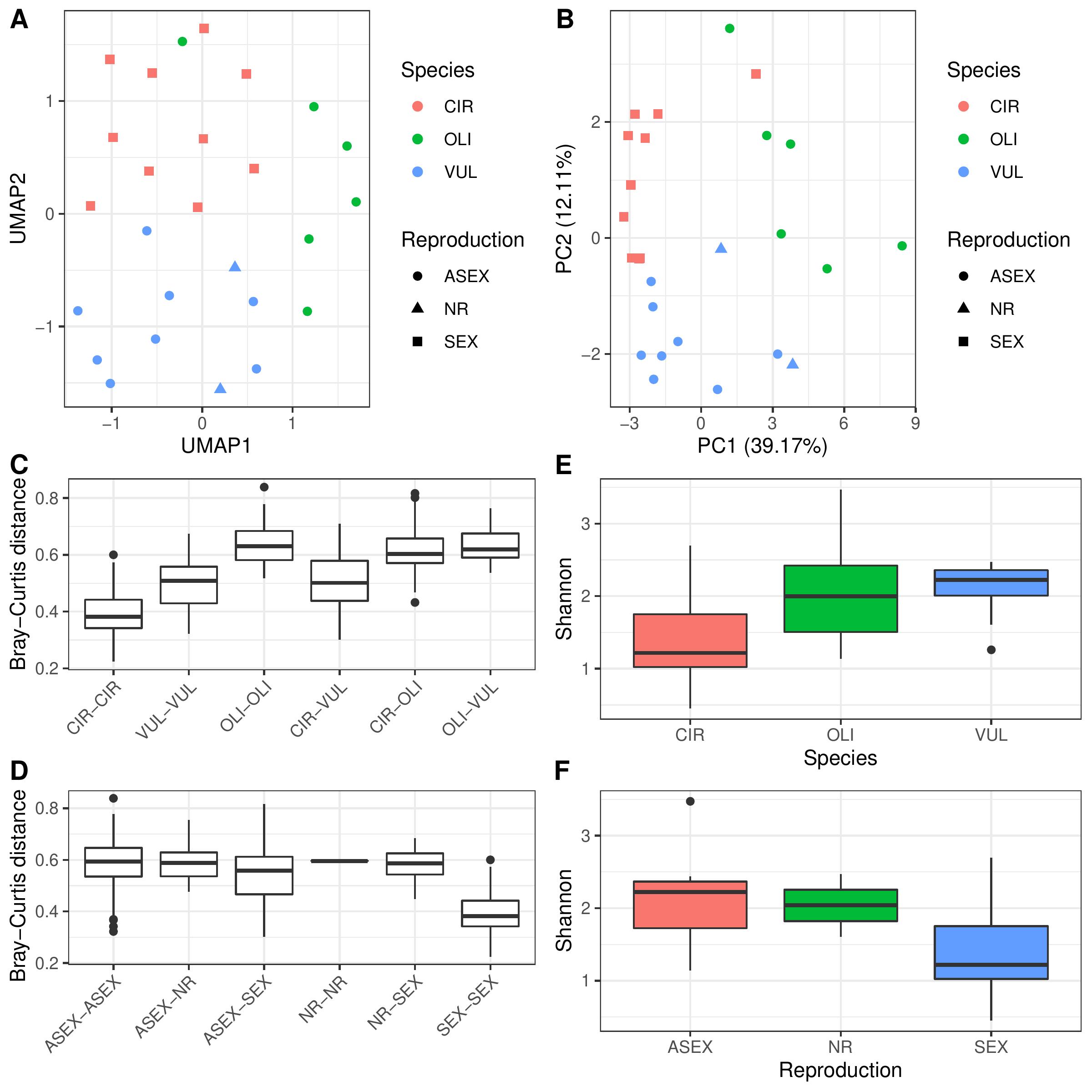

Supplement: Supplementary Figure 7 — Analysis of individual population M89 recapitulates global patterns for species associated changes in the microbiota and no influence of reproduction on microbial changes. The different species form distinct clusters in UMAP representations (A) and PCA representation (B) of β-diversity, which finds statistical support in PERMANOVA analysis (p < 0.001 in multivariate and univariate PERMANOVA respectively, Supplementary Table 3). Lower Bray–Curtis distances can be observed within H. circumcincta, H. vulgaris, and H. circumcincta—H. vulgaris pairs, while all other pairs display higher diversity changes (C)—notably also within H. oligactis pairs higher dissimilarity indices are present. There are smaller Bray–Curtis distances in sexual reproducing animals (D), which find support in univariate PERMANOVA (p = 0.004, Supplementary Table 3) but not in multivariate PERMANOVA (p = 0.59, Supplementary Table 3)—this divergence is caused by perfect collinearity of sexual reproduction and H. circumcincta thus the difference is caused by the species effect on β-diversity. α-diversity changes with different species (E) and mode of reproduction (F), but finds no statistical support in linear models (p = 0.084, p = 0.093 for the univariate and multivariate linear model and species factor, p = 0.083 and p = 0.904 for univariate and multivariate model in reproduction, Supplementary Table 4). [file Image_7.JPEG]

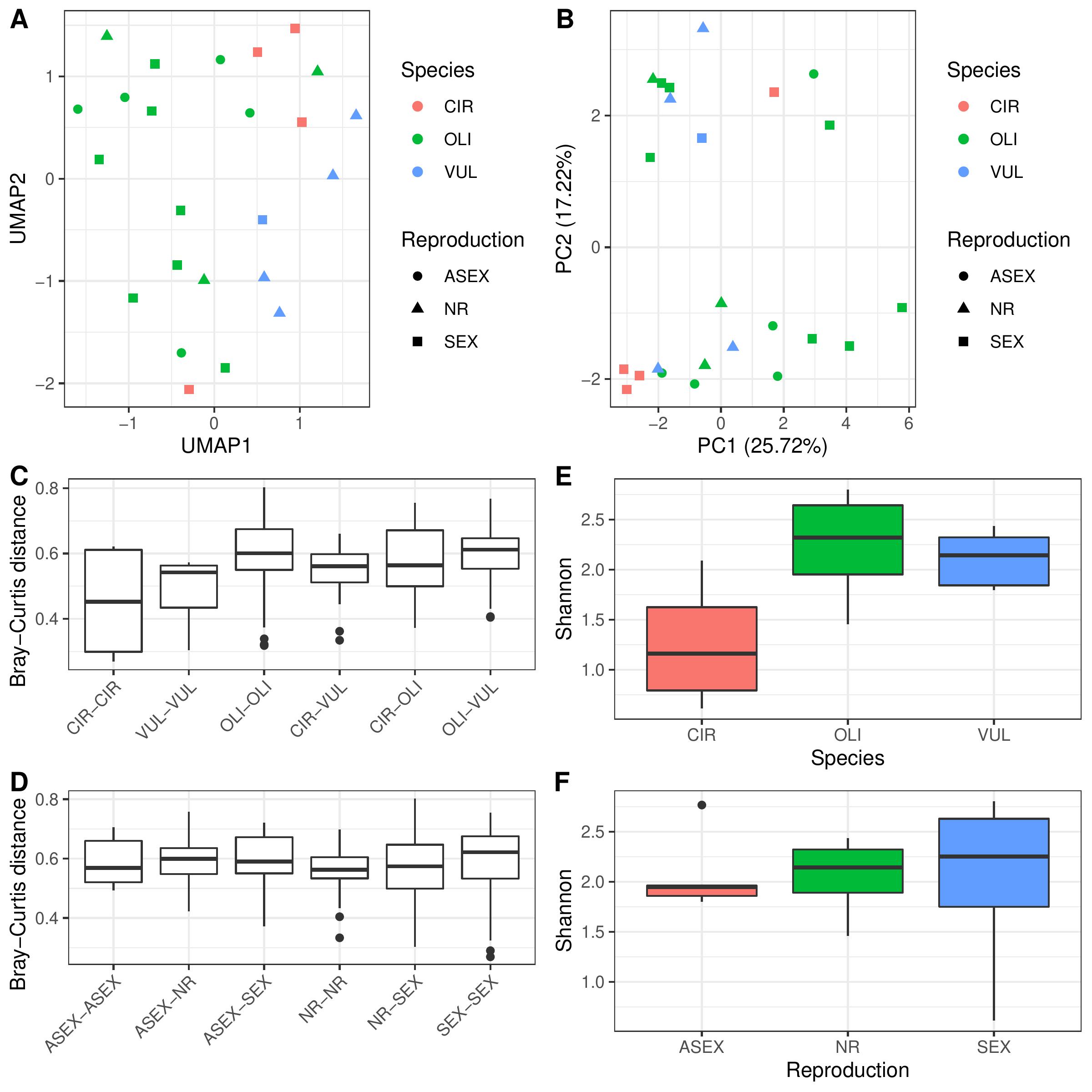

Supplement: Supplementary Figure 8 — Analysis of individual population M90 recapitulates global patterns for species associated changes in the microbiota and no influence of reproduction on microbial changes. The different species form distinct clusters in UMAP representations (A) of β-diversity, while the effect is less clear in the PCA representation (B) which finds support in statistical analysis (p < 0.001 in multivariate and univariate PERMANOVA respectively, Supplementary Table 3). Lower Bray–Curtis distances can be observed within H. circumcincta and H. vulgaris, while all other pairs display higher diversity changes (C)—notably also within H. oligactis pairs higher dissimilarity indices are present. There are no obvious differences in the Bray–Curtis distances for the different reproduction categories (D), which is also reflected in the statistical test for influence of reproduction mode on microbial diversity (p = 0.323 and p = 0.306 in univariate and multivariate PERMANOVA, Supplementary Table 3). α-diversity changes significantly with different species [(E) p = 0.002 for the univariate and multivariate linear model, respectively, Supplementary Table 4] but do not display significant differences with changes in reproductive mode [(F) p = 1 and p = 0.181 for univariate and multivariate linear model, Supplementary Table 4]. [file Image_8.JPEG]
